# Elevated plasma ceramide levels in post-menopausal women: a cross-sectional study

**DOI:** 10.18632/aging.101719

**Published:** 2019-01-08

**Authors:** Valentina Vozella, Abdul Basit, Fabrizio Piras, Natalia Realini, Andrea Armirotti, Paola Bossù, Francesca Assogna, Stefano L. Sensi, Gianfranco Spalletta, Daniele Piomelli

**Affiliations:** 1 Department of Drug Discovery and Development, Italian Institute of Technology, Genova 16163, Italy; 2 Department of Clinical and Behavioral Neurology, Laboratory of Neuropsychiatry, IRCCS Santa Lucia Foundation, Rome 00179, Italy; 3 Department of Clinical and Behavioral Neurology, Laboratory of Experimental Neuropsychobiology, IRCCS Santa Lucia Foundation, Rome 00179, Italy; 4 Department of Neurosciences, Imaging and Clinical Sciences, "G. d'Annunzio" University of Chieti-Pescara, Chieti 66100, Italy; 5 Molecular Neurology Unit, Center of Excellence on Aging and Translational Medicine (Ce.S.I.-Me.T.), "G. d'Annunzio" University of Chieti-Pescara, Chieti 66100, Italy; 6 Departments of Neurology and Pharmacology, Institute for Mind Impairments and Neurological Disorders, University of California Irvine, Irvine, CA 92697, USA; 7 Menninger Department of Psychiatry and Behavioral Sciences, Baylor College of Medicine, Houston, TX 77030, USA; 8 Departments of Anatomy and Neurobiology, Biochemistry and Pharmacology, University of California Irvine, Irvine, CA 92697, USA; * Equal contribution

**Keywords:** estrogen, menopause, sex-dependent, aging, ceramides, liquid chromatography/mass spectrometry

## Abstract

Circulating ceramide levels are abnormally elevated in age-dependent pathologies such as cardiovascular diseases, obesity and Alzheimer’s disease. Nevertheless, the potential impact of age on plasma ceramide levels has not yet been systematically examined. In the present study, we quantified a focused panel of plasma ceramides and dihydroceramides in a cohort of 164 subjects (84 women) 19 to 80 years of age. After adjusting for potential confounders, multivariable linear regression analysis revealed a positive association between age and ceramide (d18:1/24:0) (β (SE) = 5.67 (2.38); *p* = .0198) and ceramide (d18:1/24:1) (β (SE) = 2.88 (.61); *p* < .0001) in women, and between age and ceramide (d18:1/24:1) in men (β (SE) = 1.86 (.77); *p* = .0179). In women of all ages, but not men, plasma ceramide (d18:1/24:1) was negatively correlated with plasma estradiol (r = -0.294; *p* = .007). Finally, *in vitro* experiments in human cancer cells expressing estrogen receptors showed that incubation with estradiol (10 nM, 24 h) significantly decreased ceramide accumulation. Together, the results suggest that aging is associated with an increase in circulating ceramide levels, which in post-menopausal women is at least partially associated with lower estradiol levels.

## Introduction

The ceramides are key lipid constituents of mammalian cells. They regulate the structural properties of the lipid bilayer [1] along with its interaction with cellular proteins [2], and control many signalling processes, including cell survival [3], growth and proliferation [4], differentiation [5], senescence [6] and apoptosis [7,8]. Dysfunctions in ceramide-mediated signalling may contribute to the initiation and progression of a variety of age-dependent diseases. Human studies have shown the existence of abnormal plasma levels of various ceramide species – including ceramide (d18:1/18:0), (d18:1/22:0), (d18:1/24:0) and (d18:1/24:1) – in several conditions such as obesity [9], type-2 diabetes [10], hypertension [11], atherosclerosis [12] and other cardiovascular diseases [13]. Furthermore, elevated serum levels of long-chain ceramides have been linked to the increased risk of memory deficits [14] and may be predictive of hippocampal volume loss and cognitive decline in patients affected by mild cognitive impairment [15]. Other studies have reported the existence of sex-dependent differences in circulating ceramides, albeit with apparently contrasting results [16–18]. For example, in a study of a large cohort of Mexican-Americans of median age 35.7 years, plasma ceramides were found to be higher in men than in women [17]. By contrast, in the Baltimore Longitudinal Study of Aging, whose participants were aged 55 or older, plasma ceramide concentrations were shown to be higher in women than in men [18]. These discrepancies may reflect across-study differences in design and participation.

Aging in rats and mice is associated with sexually dimorphic changes in the sphingolipid composition of several brain structures, including the hippocampus [19]. Age-dependent sphingolipid alterations have also been documented in peripheral rodent tissues [20]. In the present study, we hypothesized that aging in humans might be similarly associated with changes in ceramide levels. To test this idea, we profiled six ceramides and dihydroceramide species in lipid extracts of plasma from 164 subjects (84 women) of age 19 to 80 years, using liquid chromatography/mass spectrometry (LC-MS/MS).

## RESULTS

### Plasma ceramide levels in women are positively correlated with age

**Figure 1:**
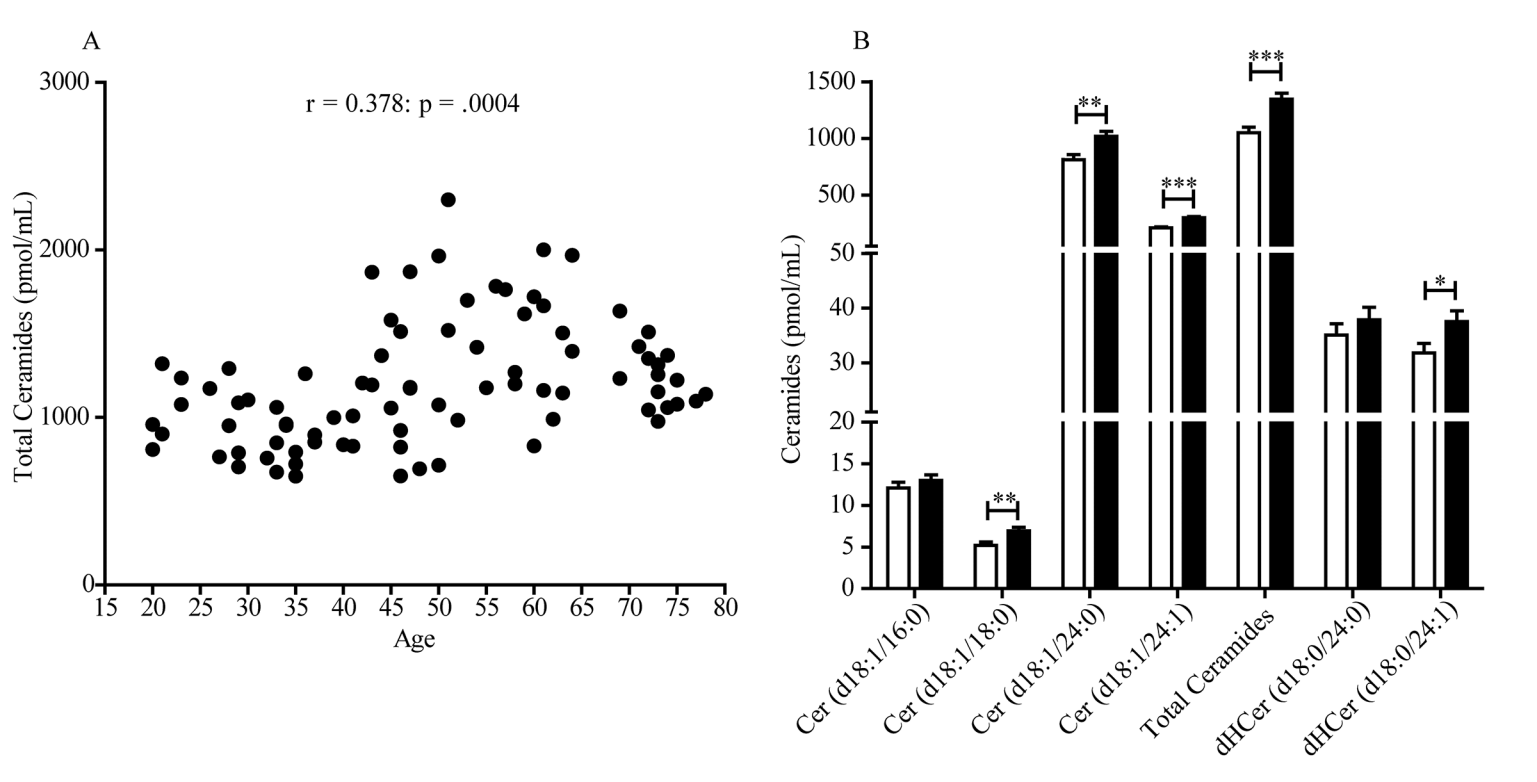
**Scatter plot of plasma ceramide concentrations in women aged 20 to 78 years.** (**A**) Total ceramide levels in 84 female subjects included in the study. Pearson’s correlation is considered statistically signiﬁcant at *p* < .05. (**B**) Average levels of individual ceramide species in pre-menopausal women (20-54 years, n = 44, open bars) and post-menopausal women (47-78 years, n = 40, closed bars). Results are expressed as mean ± SEM. *p < .05, **p < .01, ***p < .001; unpaired Student’s *t*-test.

**Figure 2:**
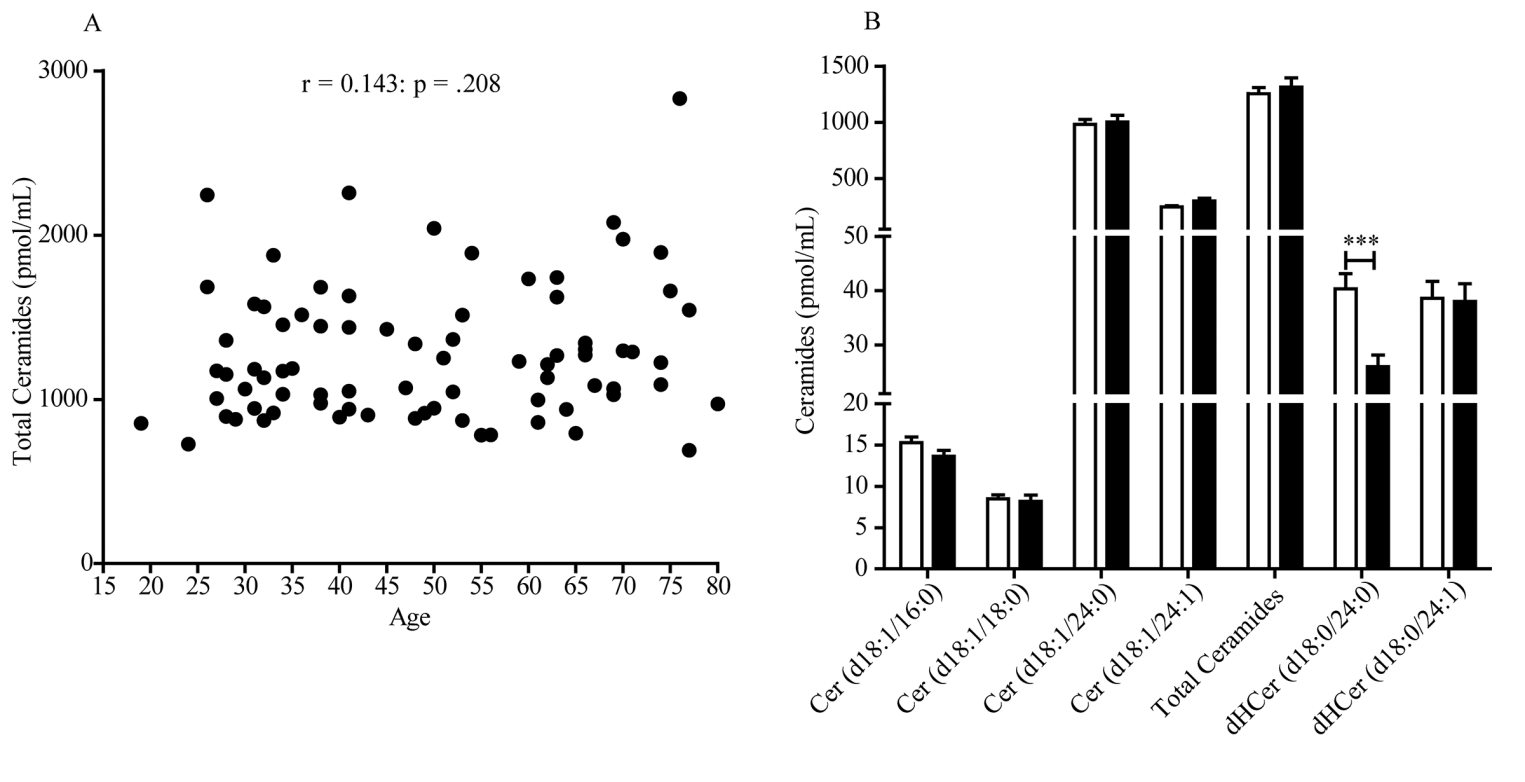
**Scatter plot of plasma ceramide concentrations in men aged 19 to 80 years.** (**A**) Total ceramide levels in 80 male subjects included in the study. Pearson’s correlation is considered statistically signiﬁcant at *p* < .05. (**B**) Average levels of individual ceramide species in men aged 19-54 years (n = 48, open bars) and 55-80 years (n = 32, closed bars). Results are expressed as mean ± SEM. *p < .05, **p < .01, ***p < .001; unpaired Student’s *t*-test.

An initial analysis of the data revealed the existence of a statistically detectable interaction between sex and ceramide levels, which prompted us to focus our attention on age-dependent changes occurring in either sex. The scatter plot reported in Fig 1A illustrates the total ceramide levels in plasma of individual women aged 20-78 years. Pearson’s analysis of the data revealed a statistically significant positive correlation between ceramide levels and age (r = 0.378; *p* = .0004). Because the largest accrual in plasma ceramides occurred between the age of 40 and 50 years, which is coincident with menopause, in a secondary analysis we grouped the data according to the subjects’ menopausal status. We found a statistically detectable difference between pre-menopausal women (20-54 years) and post-menopausal women (47-78 years) (Fig 1B). In particular, the levels of long-chain ceramide (d18:1/18:0) (*p* = .0035, unpaired Student’s *t*-test), very long-chain ceramides (d18:1/24:0) (*p* = .0012) and (d18:1/24:1) (*p* < .0001), and dihydroceramide (d18:0/24:1) (*p* = .0340) were higher in post-menopausal relative to pre-menopausal women (Fig 1B). No differences were found in the levels of ceramide (d18:1/16:0) (*p* = .3526) and dihydroceramide (d18:0/24:0) (*p* = .3633). In contrast with these findings in women, men showed no significant age-dependent increases in plasma ceramides (r = 0.143; *p* = .208) (Fig 2A). Male subjects in the age groups 19-54 and 55-80 years displayed comparable levels of circulating ceramide (d18:1/18:0) (*p* = .7112), (d18:1/24:0) (*p* = .7895), (d18:1/24:1) (*p* = .0847) and dihydroceramide (d18:0/24:1) (*p* = .9014). However, dihydroceramide (d18:0/24:0) was significantly lower in men >55 years, compared to younger men (*p* = .0003) (Fig 2B).

**Figure 3:**
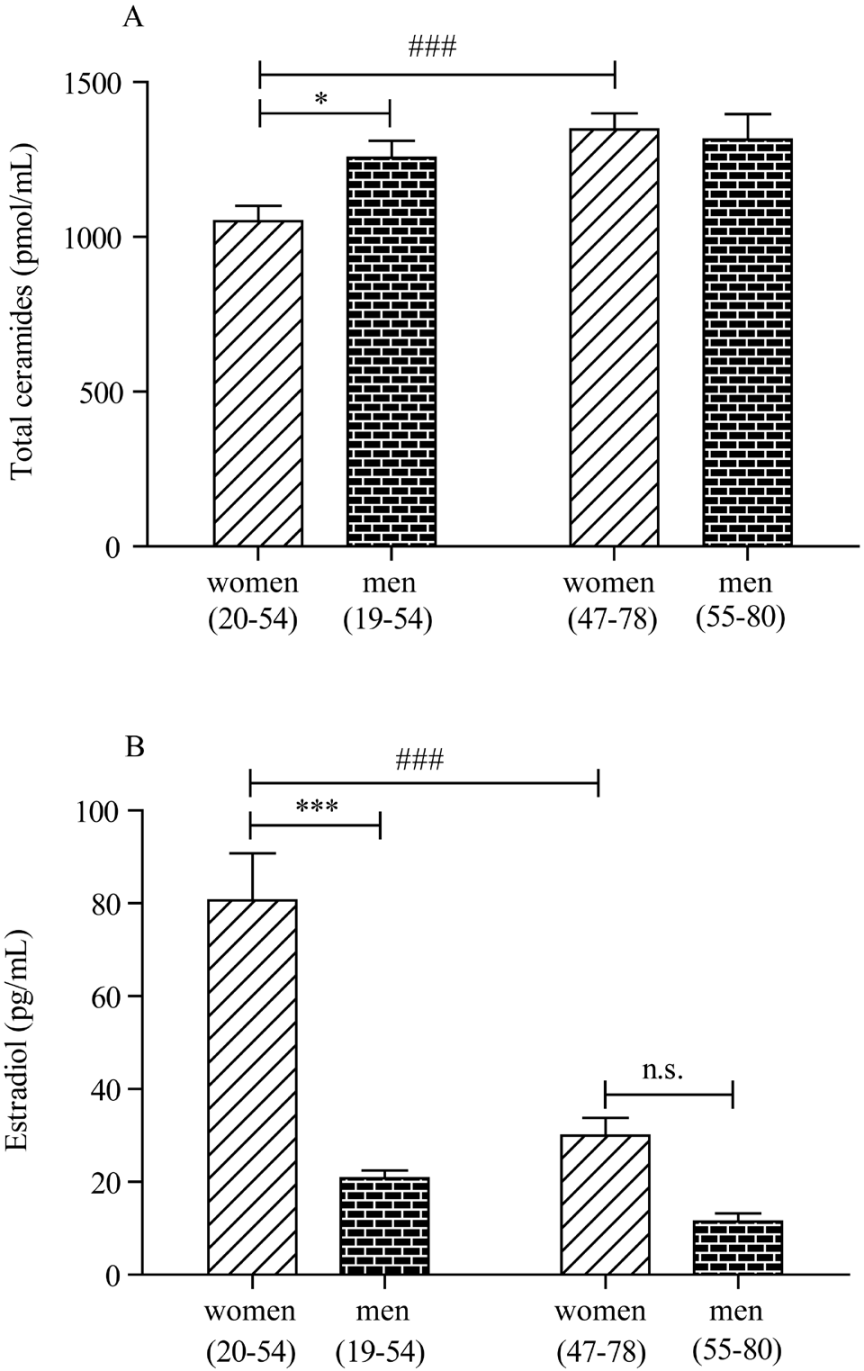
**Plasma ceramide and estradiol concentrations in men and women.** (**A**) Plasma ceramide levels in, left, pre-menopausal women (20-54 years, n = 44) and age-matched men (19-54 years, n = 48) and, right, post-menopausal women (47-78 years, n = 40) and age-matched men (55-80 years, n = 32). (**B**) Plasma estradiol levels in, left, pre-menopausal women (20-54 years, n = 44) and age-matched men (19-54 years, n = 48) and, right, post-menopausal women (47-78 years, n = 40) and age-matched men (55-80 years, n = 32). *p < .05, **p < .01, ***p < .001; 2-way ANOVA followed by Bonferroni post-hoc test (women 20-54 years versus men 19-54 years). # *p* < .05, ## *p* < .01, ### *p* < .001; 2-way ANOVA followed by Bonferroni post-hoc test (women 20-54 years versus women 47-78 years).

In an additional analysis, we compared total ceramide levels in plasma of pre- and post-menopausal women with those measured in men of the same age group (Fig 3). The results show that pre-menopausal women had significantly lower levels of circulating ceramides (*p* < .05, 2-way ANOVA followed by Bonferroni post-hoc test) relative to men of similar age (Fig 3A). The difference disappeared after menopause (*p* > .05) (Fig 3A).

### Variables associated with plasma ceramides

Next, we used multivariable linear regression models to test the association between age and ceramides and adjust for potential covariates for which data had been collected (Table 1). These factors included hypertension (32/164 subjects, 16 women), tobacco smoking (23/164 subjects, 17 women), use of anti-hypercholesterol (12/164 subjects, 3 women) or contraceptive agents (6/164 subjects, 6 women), obesity (0/164 subjects) and diabetes (0/164 subjects). We did not take into account the number of cigarettes smoked as a variable of multivariable linear regression analysis. Plasma lipid levels were not collected for these samples. The adjusted linear regression analysis confirmed that ceramide (d18:1/24:0) (β (SE) = 5.67 (2.38); *p* = .0198) and ceramide (d18:1/24:1) (β (SE) = 2.88 (0.61); *p* < .0001) were positively associated with age in women and also, unexpectedly, revealed an opposite, albeit weaker, trend with ceramide (d18:1/16:0) (β (SE) = -.08 (.04); *p* = .0285) (Table 2A). Interestingly, the analysis also pointed to a significant association, found only in women, between hypertension and ceramide (d18:1/16:0) (β (SE) = 4.78 (1.42); *p* = .0012) and (d18:1/18:0) (β (SE) = 2.48 (.97); *p* = .0126) (Table 2A). Of note, 15 out of 16 women affected by hypertension were in the post-menopausal group. In men (Table 2B), the analysis unmasked a statistically detectable association between age and ceramide (d18:1/24:1) (β (SE) = 1.86 (.77); *p* = .0179). Finally, we found no associations, in either sex, between ceramide levels and any other variable, including smoking, contraceptive use or anti-hypercholesterol agents, obesity and diabetes (Table 2A-B).

**Table 1:** Sociodemographic and clinical characteristics of men and women included in the study.

	**Whole sample** **(n=164)**	**Men** **(n=80)**	**Women** **(n=84)**	***p*-value**
**Characteristic**				
**Age** **(years)**	49.3±16.6 (range 19-80)	49.6±16.8 (range 19-80)	49.0±16.6 (range 20-78)	.46
**Education** **(years)**	14.4±3.8 (range 5-25)	14.6±4.0 (range 5-25)	14.2±3.7 (range 5-24)	.85
**MMSE**	29.4±1.0 (range 26-30)	29.4±1.0 (range 26-30)	29.4±1.0 (range 26-30)	.82
**Ethnicity** **(% white)**	100.00	100.00	100.00	
**Obesity** **(%)**	0.00	0.00	0.00	
**Diabetes** **(%)**	0.00	0.00	0.00	
**Current smokers** **(%)**	14.02	7.50	20.23	.02
**Former smokers** **(%)**	17.07	21.25	13.10	.21
**Hypertension** **(%)**	19.51	20.00	19.05	1.00
**AHT** **(%)**	7.32	11.25	3.57	.07
**Contraceptives** **(%)**	3.66	0.00	7.14	

MMSE: Mini Mental State Examination. AHT: Anti-hypercholesterol therapy. Data are expressed as mean ± standard deviation (SD) or as %. Differences between groups are considered statistically signiﬁcant at *p* < .05; unpaired Student’s *t*-test for continuous variables; Fisher’s exact test for categorical variables.

**Table 2A:** Multivariable linear regression analysis to assess the association between individual ceramide species and variables (age, smoke, hypertension, contraceptive use, anti-hypercholesterol therapy) among women.

	**Ceramide** **(d18:1/16:0)**		**Ceramide** **(d18:1/18:0)**		**Ceramide** **(d18:1/24:0)**		**Ceramide** **(d18:1/24:1)**		
	**?-coefficient (SE)**	***p*-value**	**?-coefficient (SE)**	***p*-value**	**?-coefficient (SE)**	***p*-value**	**?-coefficient (SE)**	***p*-value**	
**Covariates**									
**Age**	-0.08 (0.04)	.0285	0.01 (0.02)	.7805	5.67 (2.38)	.0198	2.88 (0.61)		<.0001	
**Smoke**	-0.22 (0.98)	.8237	0.01 (0.67)	.9846	94.84 (65.56)	.152	8.91 (16.74)		.5963	
**Hypertension**	4.78 (1.42)	.0012	2.48 (0.97)	.0126	-41.14 (94.85)	.6657	-20.60 (24.22)		.3978	
**Contraceptives**	0.26 (1.86)	.8896	-0.22 (1.27)	.8606	-200.79 (124.34)	.1104	-36.64 (31.76)		.2521	
**AHT**	5.14 (2.63)	.0544	2.76 (1.80)	.1298	-144.71 (76.10)	.4137	-51.18 (44.98)		.2586	

SE: standard error. AHT: Anti-hypercholesterol therapy. N = 84. Differences are considered statistically signiﬁcant at *p* < .05.

**Table 2B:** Multivariable linear regression analysis to assess the association between individual ceramide species and variables (age, smoke, hypertension, contraceptive use, anti-hypercholesterol therapy) among men.

	**Ceramide** **(d18:1/16:0)**		**Ceramide** **(d18:1/18:0)**		**Ceramide** **(d18:1/24:0)**			**Ceramide** **(d18:1/24:1)**			
	**?-coefficient (SE)**	***p*-value**	**?-coefficient (SE)**	***p* -value**	**?-coefficient (SE)**	***p*-value**		**?-coefficient (SE)**		***p*-value**	
**Covariates**											
**Age**	-0.05 (0.03)	.1797	-0.03 (0.03)	.3624	2.37 (2.50)		.3456		1.86 (0.77)		.0179	
**Smoke**	-0.74 (1.14)	.5199	0.25 (0.99)	.7989	-1.46 (82.89)		.986		18.25 (25.46)		.4758	
**Hypertension**	0.77 (1.35)	.5685	2.13 (1.14)	.0659	132.70 (97.13)		.176		42.58 (29.80)		.1573	
**AHT**	-2.08 (1.75)	.2388	-0.79 (1.52)	.6064	-189.47 (128.14)		.1435		-77.42 (39.38)		.053	

SE: standard error. AHT: Anti-hypercholesterol therapy. N = 80. Differences are considered statistically signiﬁcant at *p* < .05.

### Plasma ceramide levels are negatively correlated with estradiol in women, but not in men

**Figure 4:**
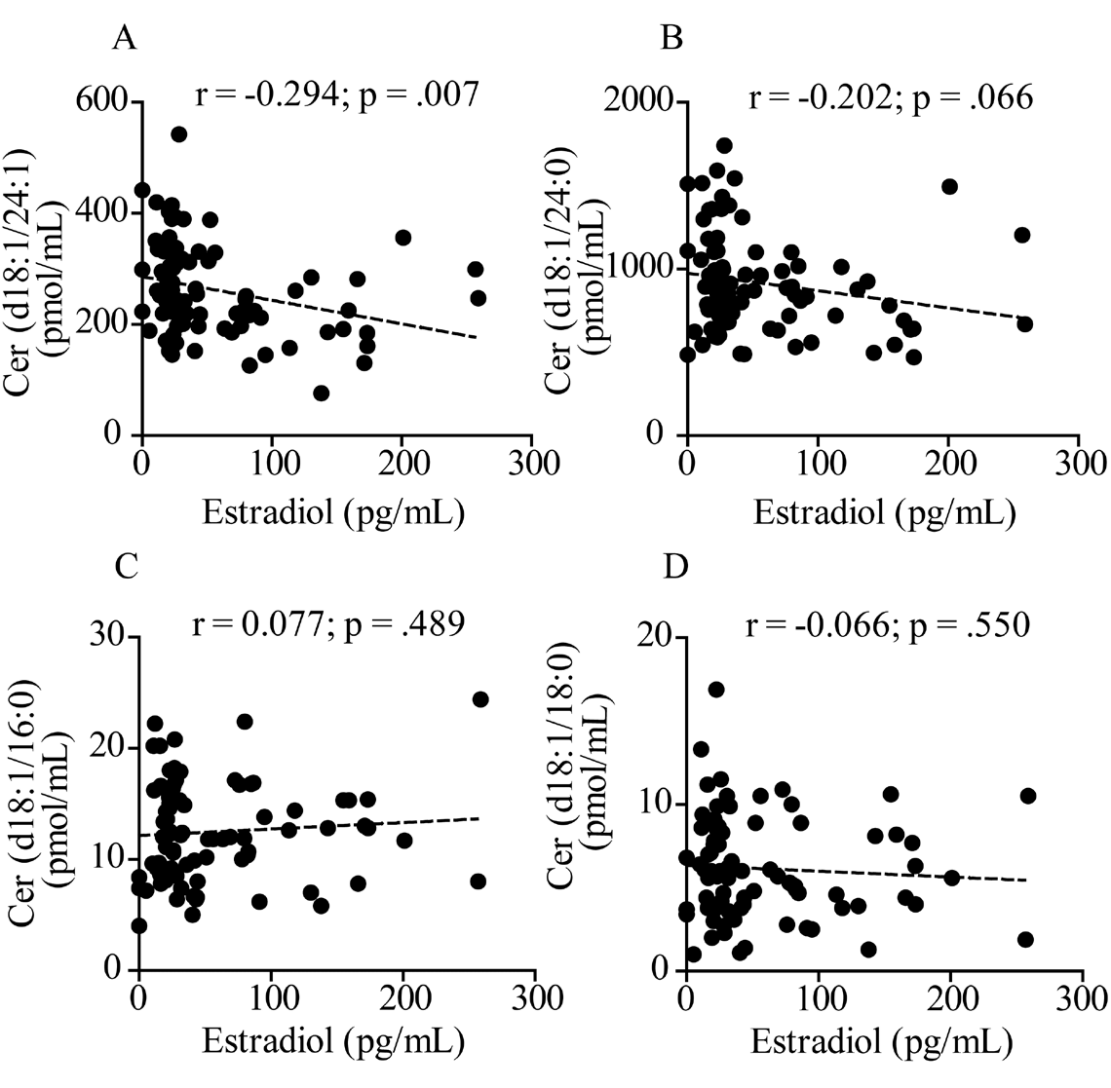
**Pearson’s correlation analysis between estradiol and levels of various ceramide species in plasma from 84 women aged 20 to 78 years.** (**A**) Ceramide (d18:1/24:1); (**B**) Ceramide (d18:1/24:0); (**C**) Ceramide (d18:1/16:0); (**D**) Ceramide (d18:1/18:0). Correlation is considered statistically signiﬁcant at *p* < .05.

**Figure 5:**
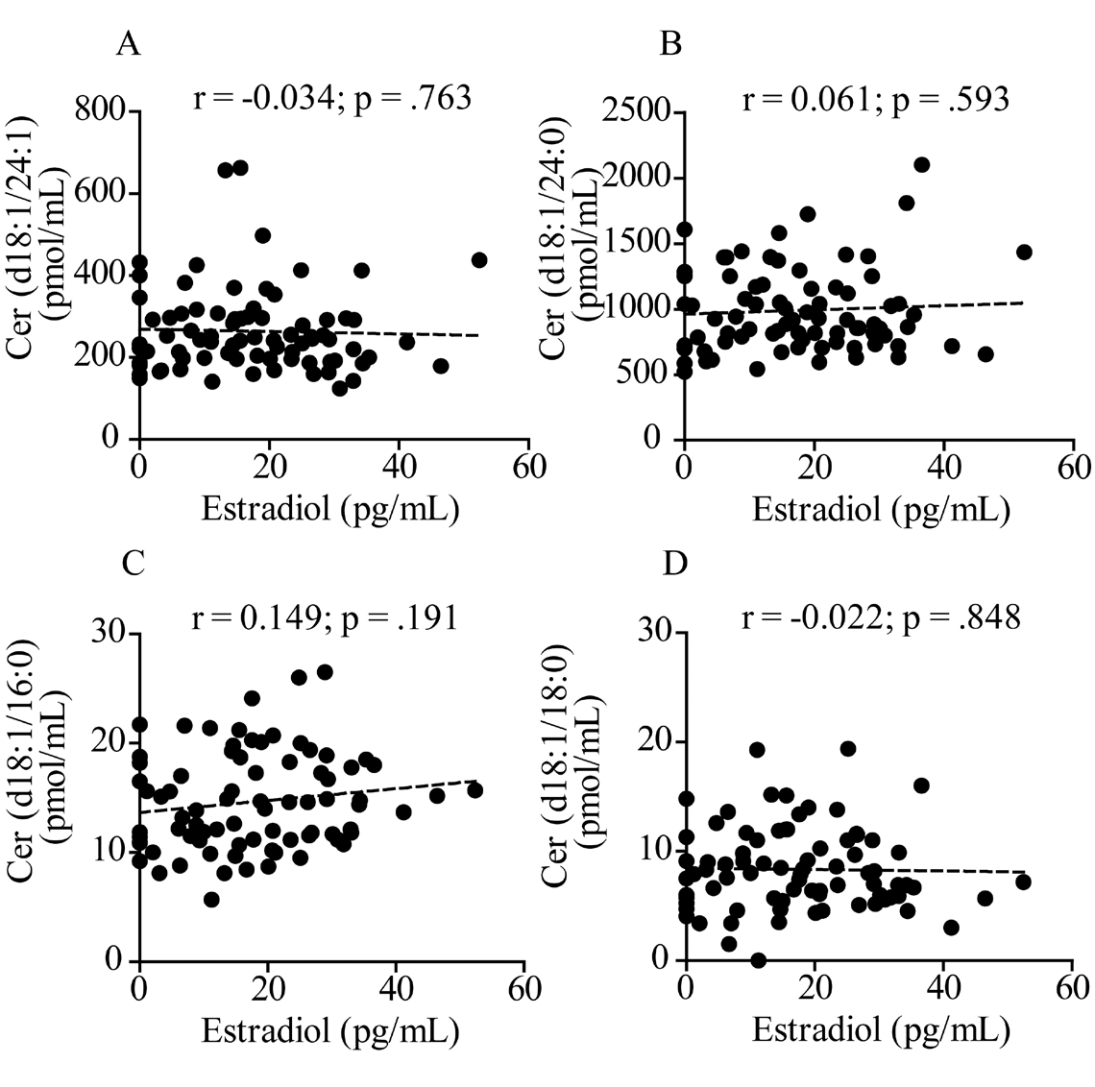
**Pearson’s correlation analysis between estradiol and levels of various ceramide species in plasma from 80 men aged 19 to 80 years.** (**A**) Ceramide (d18:1/24:1); (**B**) Ceramide (d18:1/24:0); (**C**) Ceramide (d18:1/16:0); (**D**) Ceramide (d18:1/18:0). Correlation is considered statistically signiﬁcant at *p* < .05.

Next, we investigated a possible association between plasma levels of estradiol, which are known to fall significantly at menopause, and ceramides. Estradiol was measured with a competitive binding immunoassay. As expected, plasma estradiol was higher in pre-menopausal women (<55 years) compared to men in the same age group (*p* < .001, 2-way ANOVA followed by Bonferroni post-hoc test) (Fig 3B). After menopause, estradiol levels sharply decreased in both sexes (Fig 3B). Fig 4 illustrates the results of Pearson’s analyses of ceramide levels in female subjects of all ages. The results show a statistically significant negative correlation between estradiol and ceramide (d18:1/24:1) (r = -0.294; *p* = .007), a non-significant negative trend between estradiol and ceramide (d18:1/24:0) (r = -0.202; *p* = .066) and no correlations between estradiol and other ceramide species. By contrast, in men, no correlation was observed between estradiol and any ceramide species, including ceramide (d18:1/24:1) (r = -0.034; *p* = .763) (Fig 5), which was found to be correlated with aging (β (SE) = 1.86 (.77); *p* = .0179) (Table 2B).

### Estradiol suppresses ceramide accumulation *in vitro*

Sphingolipid-derived mediators such as ceramide, sphingosine, and sphingosine-1-phosphate regulate steroidogenesis [21], but it is still unknown whether estrogen hormones influence sphingolipid metabolism. To gain insight into the causality of the negative correlation observed between estradiol and ceramide in women, we asked whether the sex hormone might regulate ceramide mobilization (i.e. formation and/or degradation) in human MCF7 breast cancer cells, a cell line that expresses the estrogen receptor α (ERα) and β (ERβ) [22]. MCF7 cells were treated with estradiol (10 nM) for 24 h and ceramides quantified in lipid extracts by LC-MS/MS. Results indicate that the exposure to estradiol causes a substantial reduction in ceramide (d18:1/16:0) (*p* = .02, unpaired Student’s *t*-test), (d18:1/24:0) (*p* = .0002) and (d18:1/24:1) (*p* = .0006) (Table 3), thereby suggesting that estradiol causes a downregulation in the biosynthesis or/and an upregulation in the degradation of these substances.

**Table 3:** Effects of estradiol on ceramide levels in MCF7 human breast cancer cells.

	**Vehicle**	**Estradiol**	***p*-value**
**Ceramide**	**(pmol/mg protein)**	**(pmol/mg protein)**	
(d18:1/16:0)	57.4 ± 6.2	41.8 ± 0.8	.02
(d18:1/18:0)	16.9 ± 1.3	13.3 ± 1.6	.12
(d18:1/24:0)	209.4 ± 14.1	134.6 ± 7.4	.0002
(d18:1/24:1)	265.6 ± 13.4	184.6 ± 12.6	.0006

Cells were treated for 24 h with vehicle (0.1% DMSO in phenol-free DMEM) or 17-β-estradiol (10 nM) and ceramide levels were measured by LC-MS/MS.

## DISCUSSION

In the present study, we investigated the age- and sex-dependent trajectories of plasma ceramides in 80 men and 84 women aged 19-80 years. The results show that, in women, plasma levels of two ceramide species – (d18:1/24:0) and (d18:1/24:1) – increased with age, and that this change cannot be ascribed to confounding factors such as obesity, diabetes, and other health conditions. In men, the analysis revealed an association between age and ceramide (d18:1/24:1). Further analyses identified a significant negative correlation between circulating levels of estradiol and ceramide (d18:1/24:1) in women of all ages, but not in men. Finally, *in vitro* experiments showed that estradiol lowers ceramide levels in human cells expressing estradiol receptors. The findings suggest that aging is accompanied, in men and women, by an increase in the plasma concentrations of two ceramide species, (d18:1/24:0) and (d18:1/24:1), which are also known to be elevated in age-dependent pathologies such as atherosclerosis and cardiovascular disease [23,24]. The results also point to the intriguing possibility that estradiol might control circulating ceramide levels in a sexually dimorphic manner.

Previous studies have reported sex-dependent differences in plasma ceramides, but with somewhat contrasting results, which might be due to disparities in sex, fasting, age and body mass index across the studies. In a small investigation of blood serum samples from 10 Caucasian volunteers (5 males aged 27-33 years and 5 females aged 26-33 years), ceramide (42:1) was found to be higher in women compared to men [16]. Another study, performed on a much larger cohort of young Mexican Americans (1,076 individuals, 39.1% males, median age 35.7 years) [17], uncovered an association between plasma ceramides and sex after adjusting for age and body mass index: ceramide levels were lower in women than in men. These disparities were mostly driven by long-chain ceramide species, such as (d18:1/22:0), (d18:1/24:0) and (d18:1/24:1). Finally, in a multiethnic population sample of 366 women and 626 men aged over 55 years, enrolled in the Baltimore Longitudinal Study of Aging, plasma ceramide concentrations were found to be higher in women compared to men [18].

These studies did not focus on age as a variable. By contrast, in the present work we set out to address the impact of aging on ceramide levels and excluded subjects with disease conditions that had been previously shown to affect ceramides, such as diabetes [25], cancer [26], renal disease [27], cardiovascular disease [28] and obesity [29]. We did not exclude subjects with hypertension, however, because the association of this disease state with altered ceramides remains to be fully established [11,18]. In our sample, we were able to confirm the presence of age- and sex-dependent differences in the plasma concentrations of certain ceramide species, but not others. A multivariable analysis showed that, in women, aging is accompanied by increased levels of ceramide (d18:1/24:0) and (d18:1/24:1). In men, the analysis also unmasked a statistically detectable association between age and ceramide (d18:1/24:1). These age-dependent changes could not be ascribed to obesity, diabetes, tobacco cigarette smoking and use of anti-hypercholesterol or contraceptive agents. Interestingly, secondary data analyses found that ceramide levels were significantly lower in female than male subjects aged 20-54, a difference that disappeared after menopause. In their 2015 study, Mielke and collaborators did not specifically include menopause as a variable, but suggested that menopause and estradiol may influence ceramide levels [18]. Two sets of results presented here support this prediction. First, we showed that, in women of all ages, plasma estradiol was negatively correlated with ceramide (d18:1/24:1) and displayed a trend toward correlation with ceramide (d18:1/24:0). No such relationship was detectable in men. Second, we found that incubation with exogenous estradiol (10 nM) lowered the intracellular levels of various ceramides, including ceramide (d18:1/24:0) and (d18:1/24:1), in human estrogen-sensitive MCF7 cells.

The findings outlined above suggest that age-dependent changes in estradiol may affect ceramide metabolism and/or transport in microsomal triglyceride transporter protein [30] differentially in men and women. The correlation between estradiol and ceramide raises the possibility that changes in the availability of certain ceramide species [e.g. ceramide (d18:1/24:1)] might be implicated in the reported cardioprotective, antihypertensive and neuroprotective effects of estradiol [31–33]. In this context, it is important to point out that increased plasma ceramide levels have been consistently linked to heightened risk of myocardial infarction and stroke [34]. Moreover, recent evidence indicates that alterations of ceramide levels may mirror, indirectly, ongoing neurodegenerative processes and might be used as a biomarker for Alzheimer’s disease development and progression [35]. Interestingly, the close association between alterations in ceramide levels and the likelihood of developing cognitive impairment and Alzheimer-related pathology may be particularly strong in women, thereby indicating that sex-related mechanisms might participate in shaping the Alzheimer phenotype. Of note, elderly women show changes in plasma ceramide levels at the onset of their memory impairment [15]. In the past few years, the neurobiology of memory disorders has received increasing attention [36]. Memory deficits are often reported by women in temporal proximity of menopause [37], and recent findings indicate distinct changes in memory processing that appear to be linked more to the post-menopausal status than to chronological aging: in peri-menopausal women, the onset and progression of cognitive decline are often associated with a menopause-related decrease in estradiol levels [38]. Thus, the interplay between estradiol and ceramides, described here, may potentially be of broad significance for a variety of age-dependent disorders, including cardiovascular disease and cognitive impairment or any intersection of the two conditions [39].

The present study has several limitations. First, even though we excluded persons affected by obesity and diabetes, we did not collect information on metabolic factors that could potentially impact ceramide mobilization – such as adiposity, physical activity and levels of cholesterol, triacylglycerol and glucose in blood [18,40–42]. Second, our study was focused on a specific subset of ceramides that we had previously found to be altered in the hippocampus of aged male and female mice [19]. This group of ceramides has been proposed as potential biomarker for cardiovascular risk [43], but is still only a small fraction of the vast number of ceramides produced by the body. Third, we measured total circulating levels of ceramides and did not attempt to separate ceramide pools bound to specific plasma lipoproteins [44]. It is possible that aging might exert an effect similar to obesity and diabetes, which have been shown to change the distribution of ceramides among lipoproteins [41].

Our findings also raise a number of relevant questions, which should be addressed in future work. First, even though the results suggest that estradiol modulates ceramide mobilization in women, the precise mechanism and functional significance of this effect remain to be determined. One possibility is that activation of estrogen receptors results in the down-regulation of *de novo* ceramide biosynthesis, for example by suppressing the expression of key enzymes such as serine-palmitoyltransferase and ceramide synthase [45]. Other possibilities are that estradiol might stimulate the expression of ceramide-hydrolyzing enzymes such as acid or neutral ceramidase or of ceramide-transporting proteins [30]. Probing the link between estradiol and ceramides will require additional experimentation, which may include measuring circulating ceramide levels in women throughout the menstrual cycle or assessing the impact of endogenous and exogenous estradiol on sphingolipid metabolism in female animals. At the functional level, studies are needed to correlate ceramide and estrogen levels to a broad panel of biomarkers (e.g., lipoprotein profile, C-reactive protein) and clinical outcome measures (e.g., future adverse cardiovascular events and cognitive impairment) [43]. Without such information, the clinical significance of our findings remains speculative. Second, the lack of correlation between estradiol and ceramide (d18:1/18:0) and lack of association with age implies that this ceramide species, though elevated after menopause, may be subjected to a different regulation than ceramide (d18:1/24:1), whose levels are correlated with estradiol and are statistically associated with age. Third, we observed a substantial age-dependent decrease in plasma dihydroceramide (d18:0/24:0) in men aged 54-80 years. The significance of this finding is presently unclear, but warrants further attention. Fourth, we unexpectedly uncovered an association between hypertension in post-menopausal women and elevated levels of ceramide (d18:1/16:0) and (d18:1/18:0). While this result is consistent with previous reports [11], caution is warranted until studies with a larger cohort of pre- and post-menopausal women are performed. Finally, as mentioned above, the relation between ceramide levels and premorbid changes in cardiovascular, metabolic or cognitive function was not investigated in our study and deserves further investigations. Despite these unanswered questions, our results reveal the existence of a link between age, estradiol and ceramides, which might contribute to age-dependent pathologies in post-menopausal women.

## MATERIALS AND METHODS

### Ethics statement

Investigation has been conducted in accordance with the ethical standards and according to the Declaration of Helsinki and according to national and international guidelines and has been approved by the authors' institutional review board.

### Study subjects

We recruited 164 Italian subjects (84 women) from 19 to 80 years of age (Table 1). A primary criterion for subject selection was the absence of major medical illnesses and, particularly, conditions that had been previously linked to ceramide alterations. A subdivision of the subjects by age and menopause status is reported in Table S1. There were no significant differences between male and female subjects with regard to age, years of education, ethnic background, cognitive status, as assessed by the Mini-Mental State Examination (MMSE), obesity and diabetes, hypertension and use of anti-hypercholesterol drugs. By contrast, there was difference in smoking status (20.23% women versus 7.5% men). Moreover, 6 of 44 pre-menopause women used contraceptives at time of enrollment. None of the post-menopause women was under hormone replacement therapy (HRT).

Exclusion criteria were: (i) suspicion of cognitive impairment or dementia based on MMSE [46] (score ≤ 26, consistent with normative data collected in the Italian population) and confirmed by a detailed neuropsychological evaluation using the mental deterioration battery [47] and clinical criteria for Alzheimer’s dementia [48] or mild cognitive impairment [49]; (ii) subjective complaints of memory difficulties or other cognitive deficits, regardless of whether or not these interfered with daily life, as assessed by clinical evaluation; (iii) vision and hearing loss that could potentially influence testing results, as assessed by clinical evaluation; (iv) major medical illnesses (i.e., unstable diabetes; obesity; obstructive pulmonary disease or asthma; hematological and oncological disorders, as assessed by clinical evaluation and pertinent clinical testing; pernicious anemia; significant gastrointestinal, renal, hepatic, endocrine, or cardiovascular system diseases, as assessed by clinical evaluation and pertinent clinical testing; recently treated hypothyroidism, as assessed by clinical evaluation; (v) current or reported psychiatric disease, as assessed by the Structured Clinical Interview for Diagnostic and Statistical Manual of Mental Disorders, 4th Edition, Text Revision (DSM-IV-TR SCID) [50] or neurological disease, as assessed by clinical evaluation; and (vi) known or suspected history of alcoholism or drug addiction. Finally, because ceramides have been previously involved in the pathogenesis of neurodegenerative disorders [15,51,52], we excluded subjects who showed brain abnormalities or vascular lesions as determined by using a recently published semi-automated method [53]. The menopausal status was prospectively assessed during clinical interviews. Women were defined as post-menopausal when showing 12 consecutive months of amenorrhea that was not due to other obvious pathological or physiological causes.

Blood collection and analyses were approved by the Santa Lucia Foundation Ethics Committee. A written consent form was signed by all participants after they received a full explanation of the study procedures. Informed consent has been obtained.

### Variables examined in relation to ceramide levels

All variables were assessed for each patient using the same methods. Demographic variables considered in linear regression analysis included age and sex. Medical history covariates included hypertension, use of anti-hypercholesterol agents and use of contraceptives. Current and former smoking status was ascertained by an oral interview.

### Chemicals

Solvents and chemicals were purchased from Sigma Aldrich (Milan, Italy). Ceramide standards were from Avanti Polar Lipids (Alabaster, AL, USA).

### Blood collection

Blood was drawn by venipuncture in the morning after an overnight fast, and collected into 10-ml tubes containing spray-coated EDTA (EDTA Vacutainer, BD Biosciences, San Diego, CA, USA). Plasma was obtained by blood centrifugation at 400 × *g* at 4 °C for 15 min. The plasma divided into aliquots was stored at −80 °C until analyses.

### Lipid extraction

Lipids were extracted using a modified Bligh and Dyer method [54]. Briefly, plasma samples (50 µL) or cell pellets were transferred to glass vials and liquid-liquid extraction was carried out using 2 mL of a methanol/chloroform mixture (2:1 vol/vol) containing the odd-chain saturated ceramide (d18:1/17:0) as an internal standard. After mixing for 30 s, lipids were extracted with chloroform (0.5 mL), and extracts were washed with liquid chromatography-grade water (0.5 mL), mixing after each addition. The samples were centrifuged for 15 min at 3500 x *g* at room temperature. After centrifugation, the organic phases were collected and transferred to a new set of glass vials. To increase overall recovery, the aqueous fractions were extracted again with chloroform (1 mL). The two organic phases were pooled, dried under a stream of N_2_ and residues were dissolved in methanol/chloroform (9:1 vol/vol, 0.07 mL). After mixing (30 s) and centrifugation (10 min, 5000 x *g*, room temperature) the samples were transferred to glass vials for analyses.

### Ceramide quantification

Ceramides were analyzed by LC-MS/MS using an Acquity® ultra-performance liquid chromatography (UPLC) system coupled to a Xevo triple quadrupole mass spectrometer interfaced with electrospray ionization (Waters, Milford, MA, USA), as previously described [54]. Lipids were separated on a Waters Acquity® BEH C18 column (2.1 × 50 mm, 1.7 μm particle size) at 60 °C and eluted at a flow rate of 0.4 mL/min. The mobile phase consisted of 0.1% formic acid in acetonitrile/water (20:80 vol/vol) as solvent A and 0.1% formic acid in acetonitrile/isopropanol (20:80 vol/vol) as solvent B. A gradient program was used: 0.0–1.0 min 30% B, 1.0–2.5 min 30 to 70% B, 2.5–4.0 min 70 to 80% B, 4.0–5.0 min 80% B, 5.0–6.5 min 80 to 90% B, and 6.6–7.5 min 100% B. The column was reconditioned to 30% B for 1.4 min. The injection volume was 3 µL. Detection was done in the positive electrospray ionization mode. Capillary voltage was 3.5 kV and cone voltage was 25 V. The source and desolvation temperatures were set at 120 °C and 600 °C respectively. Desolvation gas and cone gas (N_2_) flow were 800 L/h and 20 L/h, respectively. Plasma and cell-derived ceramides were identified by comparison of their LC retention times and MS/MS fragmentation patterns with those of authentic standards (Avanti Polar Lipids). Extracted ion chromatograms were used to identify and quantify the following ceramides and dihydroceramides (d18:1/16:0) (*m/z* 520.3 > 264.3), (d18:1/18:0) (*m/z* = 548.3 > 264.3), (d18:1/24:0) (*m/z* = 632.3 > 264.3), (d18:1/24:1) (*m/z* = 630.3 > 264.3), (d18:0/24:0) (*m/z* = 652.5 > 634.5) and (d18:0/24:1) (*m/z* = 650.5 > 632.5). Data were acquired by the MassLynx software and quantified using the TargetLynx software (Waters, Milford, MA, USA).

### Estradiol quantification

Plasma 17-β-estradiol (E2) levels were quantified using a competitive binding immunoassay kit (Human E2 ELISA kit, Invitrogen, Milan, Italy) following manufacturer’s instructions. Briefly, plasma samples, controls and standard curve samples (50 µL) were incubated with E2-horseradish peroxidase conjugate (50 µL) and anti-estradiol antibody (50 µL) in a 96-well plate for 2 h, at room temperature, on a shaker set at 700 ± 100 rpm. Washing was carried out by completely aspirating the liquid, filling the wells with diluted wash buffer (0.4 mL) provided in the kit and then aspirating again. After repeating this procedure 4 times, chromogen solution (200 µL) was added to each well; reactions were run for 15 min and stopped adding 50 µL of the stop solution provided in the kit. Absorbance was measured at 450 nm and estradiol concentrations were calculated by interpolation from the reference curve.

### Cell cultures and treatment

The MCF7 human breast cancer cell line [22,55] was a kind gift of Dr. Gennaro Colella (Mario Negri Institute, Milan, Italy). Cells were cultured in phenol red-free Dulbecco’s Modified Eagle’s Medium (DMEM) (Gibco by Life Technologies, Carlsbad, CA, USA) supplemented with charcoal-stripped fetal bovine serum (10%) (Sigma Aldrich, Milan, Italy) to starve cells from steroid hormones (starvation medium), L-glutamine (2 mM), and penicillin/streptomycin (100 µg/mL), in a humidified atmosphere (5% CO_2_, 37 °C). Cells were seeded in 6-well plates (3 x 10^5^ cells/well) and cultured for 24 h. Estradiol (Sigma Aldrich, Milan, Italy) was dissolved in dimethyl sulfoxide (DMSO) and diluted in phenol red-free DMEM to a final concentration of 10 nM (0.1% final DMSO concentration). After 24 h incubation, the media were removed, cells were washed with phosphate-buffered saline, scraped and centrifuged (800 x *g*, 4 °C, 10 min). Protein concentrations were measured using the bicinchoninic acid assay (Pierce, Rockford, IL, USA) and cell pellets were stored at -80 °C until analyses.

### Statistical analyses

Results are expressed as mean ± SEM (standard error of the mean). Sex differences in baseline demographic and health-related characteristics were examined using Fisher’s exact test for categorical variables and unpaired Student's *t*-test for continuous variables. Data were analyzed by unpaired Student's *t*-test or 2-way ANOVA followed by Bonferroni post-hoc test. Pearson’s correlation coefficient assessed the pairwise correlation between estradiol and ceramide levels. Significant outliers were excluded using the Grubbs’ test. Multivariable linear regression method was used to assess the association between ceramides and patients’ characteristics. Differences between groups were considered statistically signiﬁcant at values of *p* < .05. The GraphPad Prism software (GraphPad Software, Inc., La Jolla, CA, USA) and SAS 9.4 (SAS Institute, Cary, NC, USA) were used for statistical analyses.

## SUPPLEMENTARY MATERIAL

Table S1

## References

[1] Castro BM, Prieto M, Silva LC. Ceramide: a simple sphingolipid with unique biophysical properties. Prog Lipid Res. 2014; 54:53–67. 10.1016/j.plipres.2014.01.004 24513486

[2] Grassmé H, Riethmüller J, Gulbins E. Biological aspects of ceramide-enriched membrane domains. Prog Lipid Res. 2007; 46:161–70. 10.1016/j.plipres.2007.03.002 17490747

[3] García-Barros M, Coant N, Kawamori T, Wada M, Snider AJ, Truman JP, Wu BX, Furuya H, Clarke CJ, Bialkowska AB, Ghaleb A, Yang VW, Obeid LM, Hannun YA. Role of neutral ceramidase in colon cancer. FASEB J. 2016; 30:4159–71. 10.1096/fj.201600611R 27609772 PMC5102116

[4] Saddoughi SA, Ogretmen B. Diverse functions of ceramide in cancer cell death and proliferation. Adv Cancer Res. 2013; 117:37–58. 10.1016/B978-0-12-394274-6.00002-9 23290776

[5] Bieberich E. Ceramide and sphingosine-1-phosphate signaling in embryonic stem cell differentiation. Methods Mol Biol. 2012; 874:177–92. 10.1007/978-1-61779-800-9_14 22528448 PMC3724173

[6] Venable ME, Lee JY, Smyth MJ, Bielawska A, Obeid LM. Role of ceramide in cellular senescence. J Biol Chem. 1995; 270:30701–08. 10.1074/jbc.270.51.30701 8530509

[7] Morad SA, Cabot MC. Ceramide-orchestrated signalling in cancer cells. Nat Rev Cancer. 2013; 13:51–65. 10.1038/nrc3398 23235911

[8] Maeng HJ, Song JH, Kim GT, Song YJ, Lee K, Kim JY, Park TS. Celecoxib-mediated activation of endoplasmic reticulum stress induces de novo ceramide biosynthesis and apoptosis in hepatoma HepG2 cells mobilization. BMB Rep. 2017; 50:144–49. 10.5483/BMBRep.2017.50.3.197 28193314 PMC5422027

[9] Samad F, Hester KD, Yang G, Hannun YA, Bielawski J. Altered adipose and plasma sphingolipid metabolism in obesity: a potential mechanism for cardiovascular and metabolic risk. Diabetes. 2006; 55:2579–87. 10.2337/db06-0330 16936207

[10] Haus JM, Kashyap SR, Kasumov T, Zhang R, Kelly KR, Defronzo RA, Kirwan JP. Plasma ceramides are elevated in obese subjects with type 2 diabetes and correlate with the severity of insulin resistance. Diabetes. 2009; 58:337–43. 10.2337/db08-1228 19008343 PMC2628606

[11] Spijkers LJ, van den Akker RF, Janssen BJ, Debets JJ, De Mey JG, Stroes ES, van den Born BJ, Wijesinghe DS, Chalfant CE, MacAleese L, Eijkel GB, Heeren RM, Alewijnse AE, Peters SL. Hypertension is associated with marked alterations in sphingolipid biology: a potential role for ceramide. PLoS One. 2011; 6:e21817. 10.1371/journal.pone.0021817 21818267 PMC3139577

[12] Ichi I, Nakahara K, Miyashita Y, Hidaka A, Kutsukake S, Inoue K, Maruyama T, Miwa Y, Harada-Shiba M, Tsushima M, Kojo S, and Kisei Cohort Study Grooup. Association of ceramides in human plasma with risk factors of atherosclerosis. Lipids. 2006; 41:859–63. 10.1007/s11745-006-5041-6 17152923

[13] de Mello VD, Lankinen M, Schwab U, Kolehmainen M, Lehto S, Seppänen-Laakso T, Oresic M, Pulkkinen L, Uusitupa M, Erkkilä AT. Link between plasma ceramides, inflammation and insulin resistance: association with serum IL-6 concentration in patients with coronary heart disease. Diabetologia. 2009; 52:2612–15. 10.1007/s00125-009-1482-9 19669729

[14] Mielke MM, Bandaru VV, Haughey NJ, Rabins PV, Lyketsos CG, Carlson MC. Serum sphingomyelins and ceramides are early predictors of memory impairment. Neurobiol Aging. 2010; 31:17–24. 10.1016/j.neurobiolaging.2008.03.011 18455839 PMC2783210

[15] Mielke MM, Haughey NJ, Bandaru VV, Schech S, Carrick R, Carlson MC, Mori S, Miller MI, Ceritoglu C, Brown T, Albert M, Lyketsos CG. Plasma ceramides are altered in mild cognitive impairment and predict cognitive decline and hippocampal volume loss. Alzheimers Dement. 2010; 6:378–85. 10.1016/j.jalz.2010.03.014 20813340 PMC2933928

[16] Ishikawa M, Tajima Y, Murayama M, Senoo Y, Maekawa K, Saito Y. Plasma and serum from nonfasting men and women differ in their lipidomic profiles. Biol Pharm Bull. 2013; 36:682–85. 10.1248/bpb.b12-00799 23546298

[17] Weir JM, Wong G, Barlow CK, Greeve MA, Kowalczyk A, Almasy L, Comuzzie AG, Mahaney MC, Jowett JB, Shaw J, Curran JE, Blangero J, Meikle PJ. Plasma lipid profiling in a large population-based cohort. J Lipid Res. 2013; 54:2898–908. 10.1194/jlr.P035808 23868910 PMC3770102

[18] Mielke MM, Bandaru VV, Han D, An Y, Resnick SM, Ferrucci L, Haughey NJ. Demographic and clinical variables affecting mid- to late-life trajectories of plasma ceramide and dihydroceramide species. Aging Cell. 2015; 14:1014–23. 10.1111/acel.12369 26193443 PMC4693456

[19] Vozella V, Basit A, Misto A, Piomelli D. Age-dependent changes in nervonic acid-containing sphingolipids in mouse hippocampus. Biochim Biophys Acta Mol Cell Biol Lipids. 2017; 1862:1502-11. 10.1016/j.bbalip.2017.08.008 28855145

[20] Green CL, Mitchell SE, Derous D, Wang Y, Chen L, Han JJ, Promislow DE, Lusseau D, Douglas A, Speakman JR. The effects of graded levels of calorie restriction: IX. Global metabolomic screen reveals modulation of carnitines, sphingolipids and bile acids in the liver of C57BL/6 mice. Aging Cell. 2017; 16:529–40. 10.1111/acel.12570 28139067 PMC5418186

[21] Lucki NC, Sewer MB. The interplay between bioactive sphingolipids and steroid hormones. Steroids. 2010; 75:390–99. 10.1016/j.steroids.2010.01.020 20138078 PMC2854287

[22] Brooks SC, Locke ER, Soule HD. Estrogen receptor in a human cell line (MCF-7) from breast carcinoma. J Biol Chem. 1973; 248:6251–53. 4353636

[23] Pan W, Yu J, Shi R, Yan L, Yang T, Li Y, Zhang Z, Yu G, Bai Y, Schuchman EH, He X, Zhang G. Elevation of ceramide and activation of secretory acid sphingomyelinase in patients with acute coronary syndromes. Coron Artery Dis. 2014; 25:230–35. 24589572 10.1097/MCA.0000000000000079

[24] Yu J, Pan W, Shi R, Yang T, Li Y, Yu G, Bai Y, Schuchman EH, He X, Zhang G. Ceramide is upregulated and associated with mortality in patients with chronic heart failure. Can J Cardiol. 2015; 31:357–63. 10.1016/j.cjca.2014.12.007 25746025

[25] Galadari S, Rahman A, Pallichankandy S, Galadari A, Thayyullathil F. Role of ceramide in diabetes mellitus: evidence and mechanisms. Lipids Health Dis. 2013; 12:98. 10.1186/1476-511X-12-98 23835113 PMC3716967

[26] Kizhakkayil J, Thayyullathil F, Chathoth S, Hago A, Patel M, Galadari S. Glutathione regulates caspase-dependent ceramide production and curcumin-induced apoptosis in human leukemic cells. Free Radic Biol Med. 2012; 52:1854–64. 10.1016/j.freeradbiomed.2012.02.026 22387197

[27] Mitsnefes M, Scherer PE, Friedman LA, Gordillo R, Furth S, Warady BA, and CKiD study group. Ceramides and cardiac function in children with chronic kidney disease. Pediatr Nephrol. 2014; 29:415–22. 10.1007/s00467-013-2642-1 24389650 PMC4068150

[28] Alewijnse AE, Peters SL. Sphingolipid signalling in the cardiovascular system: good, bad or both? Eur J Pharmacol. 2008; 585:292–302. 10.1016/j.ejphar.2008.02.089 18420192

[29] Samad F, Badeanlou L, Shah C, Yang G. Adipose tissue and ceramide biosynthesis in the pathogenesis of obesity. Adv Exp Med Biol. 2011; 721:67–86. 10.1007/978-1-4614-0650-1_5 21910083

[30] Iqbal J, Walsh MT, Hammad SM, Cuchel M, Tarugi P, Hegele RA, Davidson NO, Rader DJ, Klein RL, Hussain MM. Microsomal triglyceride transfer protein transfers and determines plasma concentrations of ceramide and sphingomyelin but not glycosylceramide. J Biol Chem. 2015; 290:25863–75. 10.1074/jbc.M115.659110 26350457 PMC4646243

[31] Lagranha CJ, Silva TL, Silva SC, Braz GR, da Silva AI, Fernandes MP, Sellitti DF. Protective effects of estrogen against cardiovascular disease mediated via oxidative stress in the brain. Life Sci. 2018; 192:190–98. 10.1016/j.lfs.2017.11.043 29191645

[32] Hernández-Fonseca K, Massieu L, García de la Cadena S, Guzmán C, Camacho-Arroyo I. Neuroprotective role of estradiol against neuronal death induced by glucose deprivation in cultured rat hippocampal neurons. Neuroendocrinology. 2012; 96:41–50. 10.1159/000334229 22213775

[33] Mercuro G, Zoncu S, Piano D, Pilia I, Lao A, Melis GB, Cherchi A. Estradiol-17beta reduces blood pressure and restores the normal amplitude of the circadian blood pressure rhythm in postmenopausal hypertension. Am J Hypertens. 1998; 11:909–13. 10.1016/S0895-7061(98)00096-X 9715781

[34] Park JY, Lee SH, Shin MJ, Hwang GS. Alteration in metabolic signature and lipid metabolism in patients with angina pectoris and myocardial infarction. PLoS One. 2015; 10:e0135228. 10.1371/journal.pone.0135228 26258408 PMC4530944

[35] Mielke MM, Lyketsos CG. Lipids and the pathogenesis of Alzheimer’s disease: is there a link? Int Rev Psychiatry. 2006; 18:173–86. 10.1080/09540260600583007 16777671

[36] Jessen F. Subjective and objective cognitive decline at the pre-dementia stage of Alzheimer’s disease. Eur Arch Psychiatry Clin Neurosci. 2014 (Suppl 1); 264:S3–7. 10.1007/s00406-014-0539-z 25238934

[37] Newhouse P, Dumas J. Estrogen-cholinergic interactions: implications for cognitive aging. Horm Behav. 2015; 74:173–85. 10.1016/j.yhbeh.2015.06.022 26187712 PMC4573353

[38] Henderson VW. Action of estrogens in the aging brain: dementia and cognitive aging. Biochim Biophys Acta. 2010; 1800:1077-83. 10.1016/j.bbagen.2009.11.005 19913598

[39] Toledo JB, Arnold SE, Raible K, Brettschneider J, Xie SX, Grossman M, Monsell SE, Kukull WA, Trojanowski JQ. Contribution of cerebrovascular disease in autopsy confirmed neurodegenerative disease cases in the National Alzheimer’s Coordinating Centre. Brain. 2013; 136:2697–706. 10.1093/brain/awt188 23842566 PMC3858112

[40] Fabbri E, Yang A, Simonsick EM, Chia CW, Zoli M, Haughey NJ, Mielke MM, Ferrucci L, Coen PM. Circulating ceramides are inversely associated with cardiorespiratory fitness in participants aged 54-96 years from the Baltimore Longitudinal Study of Aging. Aging Cell. 2016; 15:825–31. 10.1111/acel.12491 27135629 PMC5013023

[41] Boon J, Hoy AJ, Stark R, Brown RD, Meex RC, Henstridge DC, Schenk S, Meikle PJ, Horowitz JF, Kingwell BA, Bruce CR, Watt MJ. Ceramides contained in LDL are elevated in type 2 diabetes and promote inflammation and skeletal muscle insulin resistance. Diabetes. 2013; 62:401–10. 10.2337/db12-0686 23139352 PMC3554351

[42] Bergman BC, Brozinick JT, Strauss A, Bacon S, Kerege A, Bui HH, Sanders P, Siddall P, Kuo MS, Perreault L. Serum sphingolipids: relationships to insulin sensitivity and changes with exercise in humans. Am J Physiol Endocrinol Metab. 2015; 309:E398–408. 10.1152/ajpendo.00134.2015 26126684 PMC4537923

[43] Summers SA. Could ceramides become the new cholesterol? Cell Metab.2018; 27:276–80. 10.1016/j.cmet.2017.12.003 29307517

[44] Hammad SM, Pierce JS, Soodavar F, Smith KJ, Al Gadban MM, Rembiesa B, Klein RL, Hannun YA, Bielawski J, Bielawska A. Blood sphingolipidomics in healthy humans: impact of sample collection methodology. J Lipid Res. 2010; 51:3074–87. 10.1194/jlr.D008532 20660127 PMC2936747

[45] Gault CR, Obeid LM, Hannun YA. An overview of sphingolipid metabolism: from synthesis to breakdown. Adv Exp Med Biol. 2010; 688:1–23. 10.1007/978-1-4419-6741-1_1 20919643 PMC3069696

[46] Folstein MF, Folstein SE, McHugh PR. “Mini-mental state”. A practical method for grading the cognitive state of patients for the clinician. J Psychiatr Res. 1975; 12:189–98. 10.1016/0022-3956(75)90026-6 1202204

[47] Carlesimo GA, Caltagirone C, Gainotti G, Fadda L, Gallassi R, Lorusso S, Marfia G, Marra C, Nocentini U, Parnetti L, and The Group for the Standardization of the Mental Deterioration Battery. The Mental Deterioration Battery: normative data, diagnostic reliability and qualitative analyses of cognitive impairment. Eur Neurol. 1996; 36:378–84. 10.1159/000117297 8954307

[48] McKhann GM, Knopman DS, Chertkow H, Hyman BT, Jack CR Jr, Kawas CH, Klunk WE, Koroshetz WJ, Manly JJ, Mayeux R, Mohs RC, Morris JC, Rossor MN, et al. The diagnosis of dementia due to Alzheimer’s disease: recommendations from the National Institute on Aging-Alzheimer’s Association workgroups on diagnostic guidelines for Alzheimer’s disease. Alzheimers Dement. 2011; 7:263–69. 10.1016/j.jalz.2011.03.005 21514250 PMC3312024

[49] Petersen RC, Morris JC. Mild cognitive impairment as a clinical entity and treatment target. Arch Neurol. 2005; 62:1160–63. 10.1001/archneur.62.7.1160 16009779

[50] First MB, Pincus HA. The DSM-IV Text Revision: rationale and potential impact on clinical practice. Psychiatr Serv. 2002; 53:288–92. 10.1176/appi.ps.53.3.288 11875221

[51] Mencarelli C, Martinez-Martinez P. Ceramide function in the brain: when a slight tilt is enough. Cell Mol Life Sci. 2013; 70:181–203. 10.1007/s00018-012-1038-x 22729185 PMC3535405

[52] Filippov V, Song MA, Zhang K, Vinters HV, Tung S, Kirsch WM, Yang J, Duerksen-Hughes PJ. Increased ceramide in brains with Alzheimer’s and other neurodegenerative diseases. J Alzheimers Dis. 2012; 29:537–47. 10.3233/JAD-2011-111202 22258513 PMC3643694

[53] Iorio M, Spalletta G, Chiapponi C, Luccichenti G, Cacciari C, Orfei MD, Caltagirone C, Piras F. White matter hyperintensities segmentation: a new semi-automated method. Front Aging Neurosci. 2013; 5:76. 10.3389/fnagi.2013.00076 24339815 PMC3857525

[54] Basit A, Piomelli D, Armirotti A. Rapid evaluation of 25 key sphingolipids and phosphosphingolipids in human plasma by LC-MS/MS. Anal Bioanal Chem. 2015; 407:5189–98. 10.1007/s00216-015-8585-6 25749796 PMC4471391

[55] Soule HD, Vazguez J, Long A, Albert S, Brennan M. A human cell line from a pleural effusion derived from a breast carcinoma. J Natl Cancer Inst. 1973; 51:1409–16. 10.1093/jnci/51.5.1409 4357757

