# Supplementary material for: Elevated plasma ceramide levels in post-menopausal women: a cross-sectional study
Source: Aging (Albany NY). 2019 Jan 8;11(1):73–88. doi: 10.18632/aging.101719 (PMC6339790; doi:10.18632/aging.101719)
Supplement: Table S1 [file aging-11-101719-s001.docx]

**Table S1. Clinical characteristics of men and women included in the study divided by age or menopause status.**

|  | **Men**  **19-54 years of age**  **(n=48)** | **Men**  **55-80 years of age**  **(n=32)** | **Women**  **pre-menopause**  **(n=44)** | **Women**  **post-menopause**  **(n=40)** |
| --- | --- | --- | --- | --- |
| **Characteristic** |  |  |  |  |
| **Current smokers**  **(%)** | 10.4 | 3.12 | 25 | 15 |
| **Former smokers**  **(%)** | 6.25 | 46.8 | 6.8 | 20 |
| **Hypertension**  **(%)** | 12.5 | 31.2 | 2.3 | 37.5 |
| **AHT**  **(%)** | 2.08 | 25 | 0 | 7.5 |
| **Contraceptives**  **(%)** | 0 | 0 | 13.6 | 0 |

AHT: Anti-hypercholesterol therapy. Data are expressed as %.
